# Fast and robust single PCR for *Plasmodium* sporozoite detection in mosquitoes using the cytochrome oxidase I gene

**DOI:** 10.1186/s12936-017-1881-1

**Published:** 2017-05-31

**Authors:** Diego F. Echeverry, Nicholas A. Deason, Victoria Makuru, Jenna Davidson, Honglin Xiao, Julie Niedbalski, Xiaoyu Yu, Jennifer C. Stevenson, Hugo Bugoro, Allan Aparaimo, Hedrick Reuben, Robert Cooper, Thomas R. Burkot, Tanya L. Russell, Frank H. Collins, Neil F. Lobo

**Affiliations:** 1 Eck Institute for Global Health, University of Notre Dame, Notre Dame, IN 46556 USA; 2 Johns Hopkins Malaria Research Institute, Johns Hopkins Bloomberg School of Public Health, Baltimore, MD USA; 3 Macha Research Trust, Choma, Zambia; 4 National Vector Borne Disease Control Programme, Ministry of Health and Medical Services, Honiara, Solomon Islands; 5 Research Department, Solomon Islands National University, Honiara, Solomon Islands; 6 Western Province Malaria Control, Gizo, Western Province Solomon Islands; 7 Australian Army Malaria Institute, Gallipoli Barracks, Enoggera 4052 Australia; 8 Australian Institute of Tropical Health and Medicine, James Cook University, Cairns, QLD 4870 Australia

**Keywords:** Malaria, *Plasmodium*, Diagnosis, Sporozoite, *Anopheles*, 18s-rRNA, Cytochrome oxidase I, Solomon Islands, Vectors, DNA barcoding

## Abstract

**Background:**

Molecular tools for detecting malaria-infected mosquitoes with improved practicality, sensitivity and specificity, and high-throughput are required. A common PCR technique used to detect mosquitoes infected with *Plasmodium* spp. is a nested PCR assay based on the 18s-rRNA gene. However, this technique has several technical limitations, is laborious and time consuming.

**Methods:**

In this study, a PCR-based on the *Plasmodium* cytochrome oxidase I (COX-I) gene was compared with the 18s-rRNA nested PCR using serial dilutions (330–0.0012 pg) of DNA from *Plasmodium vivax*, *Plasmodium falciparum* and *Plasmodium knowlesi* and with DNA from 48 positive and negative Kenyan mosquitoes (previously detected by using both ELISA and PCR). This assay for *Plasmodium* spp. DNA detection using the fast COX-I PCR assay was then performed individually on 2122 field collected mosquitoes (from the Solomon Islands) in which DNA was extracted from head and thorax.

**Results:**

The fast COX-I PCR assay took 1 h to run and consistently detected as low as to 0.043 pg of parasite DNA (equivalent to two parasites) in a single PCR, while analyses with the 18s-rRNA nested PCR required 4 h to complete with a consistent detection threshold of 1.5 pg of DNA. Both assays produced concordant results when applied to the 48 Kenyan control samples with known *Plasmodium* spp. infection status. The fast COX-I PCR identified 23/2122 *Plasmodium*-infected mosquitoes from the Solomon Islands.

**Conclusions:**

This new COX-I PCR adapted for a single PCR reaction is a faster, simpler, cheaper, more sensitive technique amenable to high-throughput analyses for *Plasmodium* DNA detection in mosquitoes and is comparable to the 18s-rRNA nested PCR. The improved sensitivity seen with the fast COX-I PCR will improve the accuracy of mosquito infection rate determination.

## Background

As worldwide malaria transmission intensity has decreased significantly over the last decade [1, 2], larger numbers of mosquitoes are required for analysis to determine accurate infection rates [3]. The ability to detect *Plasmodium* spp. sporozoites in the salivary glands of *Anopheles* species is required for malaria studies. Detecting and characterizing infective mosquitoes is necessary for vector incrimination [4], the estimation of the entomological inoculation rate [5], and when looking at transmission blocking immunity [6].

Techniques used to detect sporozoites in mosquitoes include: (i) dissection of salivary glands and examination under the microscope [7]; (ii) immunoassays to detect circumsporozoite proteins, i.e. enzyme-linked immunosorbent assays CSP-ELISA [8, 9], and rapid dipstick Immuno-Chromatographic Assays (Vec-Test™ Malaria) [10]; and (iii) PCR based assays. All three techniques have limitations in terms of practicality, sensitivity and specificity [11–13].

PCR-based methods have demonstrated a higher sensitivity for *Plasmodium* DNA detection than other methods allowing the detection of less than 10 sporozoites per µL of source material, overcoming some limitations in sensitivity in other methods used [4, 11, 14–18]. Among the molecular sporozoite detection methods, nested PCR targeting the *Plasmodium* 18s-rRNA gene is the method most extensively used [14, 15, 19] and is consequently considered the “standard” PCR [20]. In this method, a nested PCR protocol is used to first identify the presence of DNA from the *Plasmodium* genus and then up to six additional PCRs are required to identify all *Plasmodium* species causing human malaria [21]. Other methods involving the same target gene have been developed using a single multiplex PCR assay [16] and Taqman real-time PCR [4]. Recently, a PCR–RFLP was designed to target the *Plasmodium cytochrome b* mitochondrial gene [22].

The success of any PCR strategy is strongly influenced by the quality and quantity of the template, expertise of operators, stability of reagents, and can be affected by debris and carry-over from host cells or traces of reagents/template used during DNA extraction and reactions in the multi-step process [11]. Current PCR techniques may not be amenable for high throughput analysis, due to their laborious and time-consuming nature when hundreds or thousands of specimens may need to be screened for *Plasmodium* spp. infection. A fast, simple, sensitive and high-throughput method is required to improve detection of malaria infected or infective (when analyses are limited to head and thorax) mosquitoes.

Here, a new, more sensitive, and faster high-throughput PCR assay based on the *Plasmodium* cytochrome oxidase I (COX-I) gene was developed, and compared to the 18s-rRNA nested PCR method for *Plasmodium* spp. DNA detection using known positive and negative infected mosquitoes. The primary goal of this study was to provide a new molecular diagnostic tool with improved detection of malaria infections in mosquitoes both in terms of sensitivity and throughput, which can be used for malaria entomological studies and to develop and evaluate intervention strategies toward malaria control and/or elimination.

## Methods

### *Plasmodium* species reference strains and *Plasmodium* infected *Anopheles*

*Plasmodium falciparum* (HB3 strain) and *Plasmodium vivax* (Miami strain) specimens from culture (Dr. Michael Ferdig, University of Notre Dame; and BEI Resources [23] respectively) were used as reference strains. DNA from *Plasmodium* samples was extracted following directions in the E.Z.N.A. Blood DNA Mini Kit (Omega Bio-Tek, Norcross, GA). DNA of *Plasmodium knowlesi* (Malayan strain) was provided by Dr. John W. Barnwell, Centers for Disease Control and Prevention, USA. The concentration of the extracted DNA was determined using a Nanodrop 2000 (Thermo Scientific, Waltham, MA). For the validation of the new PCR, four sets of eight serial dilutions (using a 1:6 factor) for each DNA species was prepared (each by different operators), resulting in DNA concentrations of approximately 330 pg (dilution 1) to 0.0012 pg (dilution 8). Forty-eight DNA samples from Kenyan mosquitoes, infected (n = 24) and uninfected (n = 24) with *Plasmodium* spp. based on ELISA and nested-PCR (homogenized mosquito material was separated into two aliquots, to detect the CSP protein and *Plasmodium* DNA) [24, 25], were also included for PCR validation.

### Mosquito preparation and dissection

Female adult *Anopheles* mosquitoes were captured by human landing catching (HLC) by consenting village residents in Western Province, Solomon Islands (n = 2122) and preserved in 70% ethanol (Burkot et al. pers. comm.). In the laboratory, the 70% ethanol was removed and replaced by 100% ethanol for 12 h at room temperature. The ethanol was decanted and the mosquitoes (in individual tubes) were dried at 37 °C for 15 min. Dried mosquitoes were dissected under the stereoscope. The head and thorax were separated from the abdomen using sterile toothpicks and placed in a 1.5 mL microfuge tube for further processing.

### DNA extraction using a cetyltrimethylammonium bromide (CTAB)-based method

Dissected mosquito head and thorax were thoroughly ground for 20 min with a pulsating vortex mixer (VWR International, Radnor, PA) in 1.5 mL microfuge tubes containing two stainless steel beads of 3.2 mm (BioSpec Products, Inc. Bartlesville, OK) and 200 μL of 2% CTAB (Sigma-Aldrich, St Louis, MO). Samples were then incubated at 65 °C for 5 min. 200 μL of chloroform was added to each tube, the reagents were mixed, and then centrifuged at 12,000 rpm for 5 min. An isopropanol (200 µL) precipitation was performed on the transparent supernatant (at 12,000 rpm at 5 min). The centrifuged DNA pellet was washed with 70% ethanol (200 µL) and dried [26]. Each dried DNA sample was resuspended in 20 μL of PCR-grade water, gently shaken and incubated at 55 °C for 5 min. The concentration of DNA was determined using a Nanodrop 2000 and stored at −20 °C until further use.

### 18s-rRNA nested PCR

The sensitivity of the 18s-rRNA nested PCR [14, 19] (Table 1) to detect *Plasmodium* DNA was examined with serial dilutions of DNA from the reference *Plasmodium* strains and the 48 known *Plasmodium* positive and negative Kenyan *Anopheles* mosquitoes [24, 25] using the recombinant DNA polymerase (Invitrogen, Carlsbad, CA). One micro litre of DNA was used as template for nest-1 PCR and 1 µL of the resulting PCR product was used in nest-2 PCR reaction, both with a final volume of 10 μL (Table 1). Five micro litre of the nest-2 PCR product was loaded on a 1% agarose gel stained with SYBR^®^safe (Invitrogen, Carlsbad, CA) to confirm amplifications of the 235 bp product (*Plasmodium* positive) [14, 19].

**Table 1:** PCR conditions for the 18s-rRNA nested-PCR and the new COX-I PCRs for *Plasmodium* sporozoite detection

Diagnostic description	Reagents quantities and final concentration	Thermal profile
18s-rRNA genus specific PCR nest-1 [19]	1X PCR buffer, 80 μM dNTPmix, 0.8 mM MgCl_2_, 0.1 mM each primer (rPLU1–rPLU5), 0.25 U Taq polymerase	94 °C for 4 min; 35 cycles of 94 °C for 30 s, 55 °C for 1 min, 72 °C for 1 min; and 72 °C for 4 min. Time: 120 min
18s-rRNA genus specific PCR nest-2 [19]	Same as nest-1 but using rPLU3–rPLU4 primers	Same as nest-1 but annealing temperature is 62 °C. Time: 120 min
Conventional COX-I PCR	1X PCR buffer^a^, 10 mM dNTPs, 0.4 mM each primer, 1.5 mM MgCl_2_ and 0.2 µL of recombinant Taq polymerase	94 °C for 5 min; 40 cycles of 94 °C for 1 min, 62 °C for 1 min, 72 °C for 90 s; and 72 °C for 10 min. Time: 155 min
Fast COX-I PCR	1X blood phusion buffer^a^, 1 mM each primer, and 0.125 µL of blood phusion polymerase	98 °C for 4 min; 70 cycles of 98 °C for 1 s, 69 °C for 5 s, 72 °C for 35 s; and 72 °C for 10 min. Time: 62 min

^a^The Blood Phusion buffer contains MgCl_2_ at a final concentration of 3 mM

### Single step PCR for *Plasmodium* sporozoite detection based on the cytochrome oxidase I

The nucleotide sequences of human-*Plasmodium* species (*P. falciparum*, *P. vivax*, *P. knowlesi*, *Plasmodium malariae*, *Plasmodium ovale wallikeri* and *Plasmodium ovale curtisi*) cytochrome oxidase I (COX-I), contained in the mitochondrial genome, were downloaded from GeneBank [27] and aligned as described previously [28]. A set of primers, COX-IF (5′ AGAACGAACGCTTTTAACGCCTG 3′) and COX-IR (3′ ACTTAATGGTGGATATAAAGTCCATCCwGT 5′), were designed to amplify a polymorphic fragment in the COX-I gene (DNAstar Lasergene^®^ 11 software, DNAstar Inc. Madison, WI). Two master-mixes were prepared, one using a recombinant DNA polymerase (Invitrogen, Carlsbad, CA) in 25 µL of PCR reaction (named the conventional COX-I PCR) (Table 1) and another prepared using the Blood Phusion polymerase (Thermo Scientific, Waltham, MA) with 15 μL of PCR reaction (named the fast COX-I PCR) (Table 1) using 2 µL of DNA template.

The sensitivity of COX-I PCRs (conventional and fast) to detect *Plasmodium* DNA was evaluated using the DNA (serial dilutions) of reference strains and the 48 control samples from Kenya [24, 25]. Five micro litre of the PCR product was visualized on 1% agarose gel in order to confirm amplifications of the expected ~540 bp product (*Plasmodium* genus positive). Differences between the performance of the 18s-rRNA nested PCR and the COX-I PCRs were evaluated with the McNemar’s Chi Square test.

### Fast COX-I PCR sequencing reaction

The PCR sequencing reactions were performed as previously described [28]. In brief, 8 μL of PCR product from the fast COX-I PCR was purified, and a sequencing-PCR performed with the COX-IF primer (Table 1). Samples were sequenced on an ABI 3730XL 96-capillary sequencer. Sequence analyses were performed using the DNASTAR Lasergene ^®^ 11 software (DNAstar Inc. Madison, WI).

## Results

### Comparison of COX-I PCRs and 18s-rRNA nested-PCR using DNA (serial dilutions) of *Plasmodium* reference strains

**Fig. 1:**
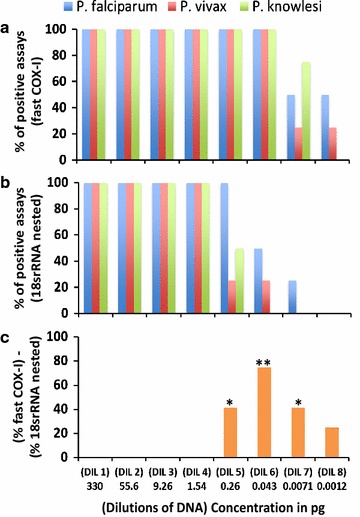
*Plasmodium* spp. detection using the fast COX-I PCR and the 18s-rRNA nested-PCR. **a** Percentage of successful *Plasmodium* DNA detection in four different assays using the fast COX-I PCR. **b** Percentage of successful *Plasmodium* DNA detection in four different assays using the 18s-rRNA nested PCR. **c** Percentage of *Plasmodium* DNA detected by COX-I PCR but not by the 18s-rRNA nested PCR based on 12 different assays; *p* value (McNemar’s Chi square test) = 0.036 (*) and 0.0038 (**). The predicted number of parasites based on Li et al. [32], in dilutions 1–8 were 15,348, 2586, 430, 71, 12, 2, 0.33 and 0.05 parasites respectively

The DNA extracted with the E.Z.N.A. Blood DNA Mini Kit from *P. vivax* and *P. falciparum* reference strains were 30 and 7 ng/µL (OD_260_:OD_280_ average of 1.93) respectively. The DNA of *P. knowlesi* was provided at 20 ng/µL. The performance of the fast COX-I PCRs (the conventional COX-I PCR results were not shown as they were similar to the fast COX-I PCR) and the 18s-rRNA nested PCR method for *Plasmodium* spp. detection were compared using eight serial dilutions (Fig. 1). For the fast COX-I PCR, consistent and successful amplifications (100% of positivity) were achieved for all parasite species down to dilution 6 (0.043 pg), followed by 25–75% of positivity for all parasites in dilution 7, and 25–50% of positivity of *P. vivax* and *P. falciparum* in the final dilution (0.0012 pg) (Fig. 1a).

In contrast, the 18s-rRNA nested PCR detected consistent amplifications of all DNA parasites down to dilution 4 (1.5 pg of DNA). The parasite positivity rate in dilutions 5, 6, and 7, was variable, while no PCR amplification was detected in the final dilution (Fig. 1b). Taking into account all the repetitions (12 repetitions using three operators for each dilution), both fast COX-I PCR and the 18s-rRNA nested PCRs detected parasites down to the fourth dilution (1.54 pg), however, for dilutions 5, 6, and 7, the percentage of *Plasmodium* DNA detected by fast COX-I was significantly higher when compared with the 18s-rRNA (P value ≤ 0.036) (Fig. 1c).

**Fig. 2:**
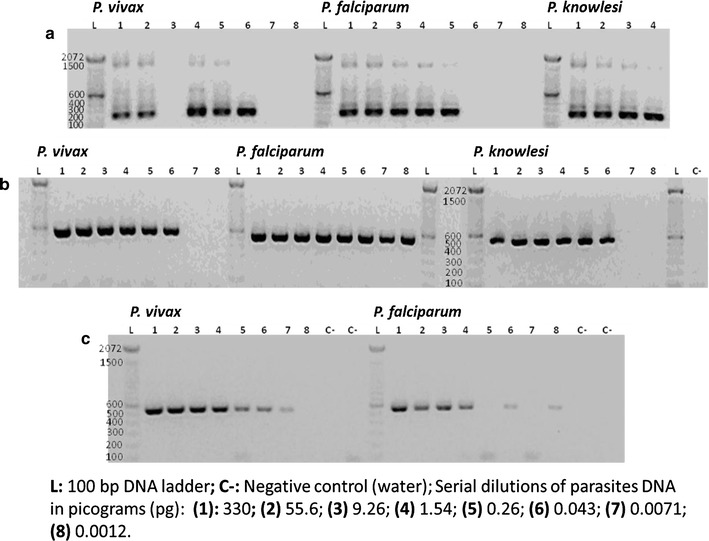
Electrophoresis gels of PCR products obtained from the 18s-rRNA nested-PCR and COX-I PCRs using serial dilutions of parasite DNAs. **a** PCR products (235 bp) from nest 2 PCR using the 18s-rRNA nested PCR. Dilution 3 for *P. vivax* did not amplified; PCR products for dilutions 5–8 for *P. knowlesi* were negatives and not included in this figure. **b** PCR products (540 bp) from the fast COX-I PCR. The PCR product bands have consistent size and intensity through all the positive dilutions. **c** PCR products (540 bp) from the conventional COX-I PCR. This PCR was not tested in *P. knowlesi*. Bands for *P. falciparum* did not amplify consistently

The expected bands of 235 bp (for the 18s-rRNA nested PCR) (Fig. 2a) and 540 bp (for the fast COX-I PCR) (Fig. 2b), were robust and no significant loss of intensity was seen between dilutions down to the last positive amplification. The conventional COX-I PCR showed bands with lower intensity and inconsistencies after dilution 4 (Fig. 2c) when compared to the fast COX-I PCR (Fig. 2b). Non-specific amplification was seen with the 18s-rRNA protocol but none with the COX-I PCRs (Fig. 2). Finally, PCR products (from the fast COX-I PCR) of *P. vivax*, *P. falciparum,* and *P. knowlesi* (all from dilution 6) were successfully sequenced and their identity confirmed (Table 2).

**Table 2:** Summary of sequenced DNA samples (positive controls, Kenya and Solomon Islands) based on *Plasmodium* COX-I gene

Sample	Species ID^a^	E value^b^	Coverage^c^ (%)	Identity^d^	[GenBank identifier]^e^
PCR product from dilution 6 (0.043 pg of DNA)					
*P. falciparum*	*P. falciparum*	0	100	99.7%	[KM065500.1]
*P. vivax*	*P. vivax*	1.3e–84	100	98.4%	[KF668441.1]
*P. knowlesi*	*P. knowlesi*	8e–180	100	100%	[AB444108.1]
Known infective mosquitoes from the field (Kenya Highlands)					
Mosquitoes (n = 21)	*P. falciparum*	0	100	99.7%	[KM065500.1]
Mosquitoes (n = 3)	*P. ovale s.l.*	1.21e–151	100	97.5%	[KF018660.1]
Positive mosquitoes from Western Province, Solomon Islands					
Mosquitoes (n = 17)	*P. falciparum*	0	100	99.7%	[KM065500.1]
Mosquitoes (n = 5)	*P. vivax*	0	99	99.6	[KF668441.1]
Mosquito (n = 1)	*P. ovale wallikeri*	7.1e–110	100	92.2%	[HQ712053.1]

^a^Best BLASTed hit in GeneBank

^b^Probability of observing the result by chance

^c^Percentage coverage of entire sequence against best hit

^d^Percentage of similarity against best hit

^e^GeneBank ID of the best hit

### Sensitivity and specificity between 18s-rRNA nested PCR and fast COX-I PCR for *Plasmodium* detection

DNA samples (n = 48) obtained from known infected and uninfected mosquitoes from Kenya [24, 25] were used to validate the new COX-I PCR assays. All known infected samples (n = 24) were positive for *Plasmodium* species using the 18s-rRNA nested PCR and the fast COX-I PCR (100% sensitivity), while 22/24 of the samples were positive with the conventional COX-I PCR assay (92% sensitivity). All 24 uninfected samples were negative when using the three techniques (100% specificity). The PCR products from the fast COX-I PCR were sequenced and database comparisons demonstrated that 21 samples were *P. falciparum* and 3 samples were *P. ovale s.l*. (Table 2).

### Presence of *Plasmodium* infective mosquitoes from the Solomon Islands

The DNA (head and thorax) of 2122 wild-caught anopheline mosquitoes from the Solomon Islands, was extracted using a CTAB-based method and screened for the presence of *Plasmodium* DNA using the fast COX-I PCR. An average of 47.89 μg/μL (range 10.6–150.2) of DNA was obtained, with an average OD_260_:OD_280_ of 2.01 (range 1.7–2.3). Twenty-three samples were positive for *Plasmodium* DNA in *Anopheles farauti* mosquitoes. Seventeen were positive for *P. falciparum*, five for *P. vivax,* and one for *P. ovale wallikeri* (Table 2).

## Discussion

The excess of host DNA may interfere with the performance of *Plasmodium* PCR diagnosis. The DNA from *P. falciparum* (HB3 strain) was pure as was obtained from long-term culture, while DNA from the *P. vivax* (Miami strain) and *P. knowlesi* (Malayan strain) was a mix of parasite and primate hosts DNA (as there is not a long-term in vitro culture available for these species). The presence of host DNA may explain the lower sensitivity of the 18s-rRNA for *P. vivax* and *P. knowlesi* in dilution 5 and 6 (Fig. 1a), however this limitations was overcome by the fast-COX-I PCR.

The fast COX-I PCR consistently detected down to 0.043 pg of DNA (dilution 6), equivalent to two parasites [29], which is >460-fold more sensitive for *Plasmodium* DNA detection than other PCR techniques (Table 3) [4, 18, 22, 30]. This may be explained by both the higher number of the COX-I gene copies (up to 150) while 18s-rRNA has only eight or fewer [31], the well-designed COX-I primers and the use of the Blood Phusion polymerase, a proofreading polymerase with a processivity-enhanced domain [32] that performs in the presence of strong PCR inhibitors, including collagen and melanin, compounds of the insect cuticle [33]. An infected mosquito can carry several thousand down to seven sporozoites of *Plasmodium* spp. in their salivary glands [34] suggesting that the fast COX-I PCR is sufficient for identifying infective mosquitoes.

**Table 3:** Summary of other PCR techniques for *Plasmodium* sporozoite detection

Molecular sporozoite detection approach [ref]	DNA extraction	*Plasmodium* species	Cycling time in min	DNA limit of detection
18s-rRNA nested PCR protocol [4, 15]	Livak or DNAzol methods	*P. vivax, P. falciparum, P. ovale, P. malariae*	294	0.2 ng–0.2 pg
18s-rRNA single PCR [4, 15]		*P. vivax, P. falciparum, P. ovale, P. malariae*	205	2 ng–4 pg
18s-rRNA Taqman assay [4]		*P. falciparum, P. ovale, P. malariae, P. vivax*	47	0.2 pg
18s-rRNA single PCR Tassanakajon [4, 18]		*P. falciparum*	60*	2 pg
Cytochrome B single PCR [22]	IsoQuick nucleic acid extraction kit	*P. vivax, P. falciparum*	96	0.2 pg
DHFR-TS nested [30]	Chelex	*P. falciparum*	>294	4–40 pg
Fast COX-I single PCR [this manuscript]	CTAB	*P. vivax, P. falciparum, P. ovale s.l*.*, P. knowlesi, P. ovale wallikeri*	62	0.043 pg

*min* minutes, *ng* nanograms, *pg* picograms

* The original paper from Tassanakajon et al. [18] did not include times for denaturation and final extension

The cycling time for the fast COX-I PCR, is completed in an hour, a shorter time than the other techniques (Table 3). This will enable the processing of larger quantities of samples in shorter periods of time reducing processing time and costs. The PCR cost of processing 2122 DNA samples for *Plasmodium* spp. using the 18s-rRNA nested PCR or the conventional COX-I PCR is ~892 USD, while for the fast COX-I PCR is ~552 USD (Table 4). The fast COX-I PCR minimizes the risk of contamination and amplification of non-specific bands—the two primary technical limitations in nested PCR strategies or when DNA was derived from mosquitoes stored in ethanol or isopropanol [4, 35]. This will be particularly important when looking at vector incrimination or large numbers of mosquito samples where infection rates might be low such as with secondary vectors or vectors with low vectorial capacity.

**Table 4:** Summary of cost analysis for the 18s-rRNA nested and COX-I PCRs for *Plasmodium* spp. detection

PCR technique	Required PCR reagents	Estimated cost of the PCR kit	µL of polymerase used per reaction	Cost of PCR diagnosis per sample	Cost of 2122 reactions for *Plasmodium* detection
18s-rRNA nested PCR	Taq polymerase kit (Invitrogen)	~210 USD	0.1 µL for nest-1 and 0.1 µL nest-2	~0.42 USD	~892 USD
Conventional COX-I single PCR	Taq polymerase kit (Invitrogen)	~210 USD	0.2 µL in a single reaction	~0.42 USD	~892 USD
Fast COX-I single PCR	Blood Phusion polymerase kit (Thermo)	~418 USD	0.125 µL in a single reaction	~0.26 USD	~552 USD

For a set of 24 known *Plasmodium* positive mosquitoes [24, 25], all PCRs were positive with the fast COX-I PCR, which confirms that the new PCR is able to detect *Plasmodium* DNA in samples from the field. The conventional PCR, which uses a recombinant DNA polymerase and the same primers (Table 1), did not amplify 2/24 of the positive samples. This may be explained by low parasite DNA quality or quantity and/or presence of PCR inhibitors in the samples. In either case, the fast COX-I PCR was able to overcome these limitations and identified these samples as positives (*P. falciparum*).

The fast COX-I PCR was successfully tested in different anopheline species with different human malaria parasites. The *Anopheles* mosquitoes tested included *Anopheles farauti*, *Anopheles hinesorum*, *Anopheles lungae*, and *Anopheles solomonis* from the Solomon Islands, and *Anopheles funestus*, *Anopheles coustani*, *Anopheles maculipalpis*, *Anopheles theileri,* and *Anopheles leesoni* amongst others from Kenya, suggesting that this PCR can be used across vector species. This PCR-sequencing approach functioned across human *Plasmodium* species including *P. knowlesi.* The COX-I primers had 100% of identity and 100% coverage with at least 26 different *Plasmodium* species including parasites from lizards, birds, rodents and non-human primates, which may be relevant in assessing malaria transmission in particular settings (e.g. forest border areas). At the core of this technique (DNA barcoding), the use of COX-I relies in the use of a set of primers that recognize a flanking conserved region for *Plasmodium* spp. surrounding an internal variable region that allows species identification by sequencing of the amplified fragment.

## Conclusion

The fast COX-I PCR designed for *Plasmodium* species sporozoite detection is more sensitive, less expensive, and faster than other PCR strategies utilized at present. This functionally better diagnostic may be utilized in both research, intervention strategies and monitoring studies towards identifying infected and infective mosquitoes.

## Abbreviations

CSP-ELISA: circumsporozoite protein-enzyme-linked immunosorbent assay
COX-I: cytochrome oxidase I
18s-rRNA: 18 small subunit ribosomal RNA
BEI Resources: the biological and emerging infections resources program
min: minute
sec: second
µL: microlitre
pg: picogram
ng: nanogram
